# Effects of processing on structural, mechanical and biological properties of collagen-based substrates for regenerative medicine

**DOI:** 10.1038/s41598-018-19786-0

**Published:** 2018-01-23

**Authors:** A. Terzi, E. Storelli, S. Bettini, T. Sibillano, D. Altamura, L. Salvatore, M. Madaghiele, A. Romano, D. Siliqi, M. Ladisa, L. De Caro, A. Quattrini, L. Valli, A. Sannino, C. Giannini

**Affiliations:** 1 Institute of Crystallography (IC), National Research Council, Bari, Italy; 2 Department of Engineering for Innovation, University of Salento, Lecce, Italy; 3 Neuropathology Unit, Institute of Experimental Neurology and Division of Neuroscience, IRCCS San Raffaele Scientific Institute, Milan, Italy; 4 Department of Biological and Environmental Sciences and Technologies, University of Salento, Lecce, Italy

## Abstract

The aim of this work was to investigate the structural features of type I collagen isoforms and collagen-based films at atomic and molecular scales, in order to evaluate whether and to what extent different protocols of slurry synthesis may change the protein structure and the final properties of the developed scaffolds. Wide Angle X-ray Scattering data on raw materials demonstrated the preferential orientation of collagen molecules in equine tendon-derived collagens, while randomly oriented molecules were found in bovine skin collagens, together with a lower crystalline degree, analyzed by the assessment of FWHM (Full Width at Half Maximum), and a certain degree of salt contamination. WAXS and FT-IR (Fourier Transform Infrared) analyses on bovine collagen-based films, showed that mechanical homogenization of slurry in acidic solution was the treatment ensuring a high content of super-organization of collagen into triple helices and a high crystalline domain into the material. ***In vitro*** tests on rat Schwannoma cells showed that Schwann cell differentiation into myelinating cells was dependent on the specific collagen film being used, and was found to be stimulated in case of homogenization-treated samples. Finally DHT/EDC crosslinking treatment was shown to affect mechanical stiffness of films depending on collagen source and processing conditions.

## Introduction

Type I collagen is the main protein of the mammalian extracellular matrix (ECM), providing structural stability and strength. In each tissue, collagen molecules are structured and arranged in a very specific way, which depends on the biological and mechanical performance of the tissue itself^1,2^.

From a molecular point of view, type I collagen is a hetero trimer which consists of two α1 chains, encoded by COL1A1, and one α2 chain, encoded by COL1A2^3^. More in detail, the single collagen chain comprises three parts: two short non-helical regions at both the N- and C-termini, and a long central helical part that contains 1014 amino acid residues, with a strict repetition of the Gly-X-Y triplet that enables the triple-helical conformation. The X and Y positions are occupied by two imino acids, typically Pro and Hyp respectively, that are exposed on the surface and can sterically interact with any amino acid. Gly is buried at the center of the triple helix. The preservation of this amino acid in every third position of the triplet is required for close packing of the three helices that constitute the well-known triple helix structure^4^.

The triple helix is a rod-like structure stabilized by hydrogen bonding, either direct (between the backbone NH group of glycine and the backbone CO group of a residue in the X position of the neighboring chain) or water mediated. Water bridges between different chains and between different triple helices are formed. The water molecules bridge and surround the triple helices in the collagen crystal lattice with a cylinder of hydration^5^. Indeed, at concentration above 30–40 mg/ml collagen molecules self-assemble in liquid crystal phases corresponding to nematic (directional, not layered molecules) and cholesteric (directional molecules with a layered helical pattern) geometries.

In the last 60 years, X-ray scattering measurements on collagen-based connective tissues allowed to characterize the triple helix structure. Wide Angle X Ray Scattering (WAXS) technique permits to analyze the interference pattern of the secondary waves scattered by the atomic electron density distribution within the crystalline structure of the triple helices, in order to obtain structural information at the atomic scale. X-ray scattering indeed allows identifying the presence of three chains in the polyproline II conformation, supercoiled around each other with a periodicity of 2.9 Å in the direction of the helical axis^6^.

**Figure 1:**
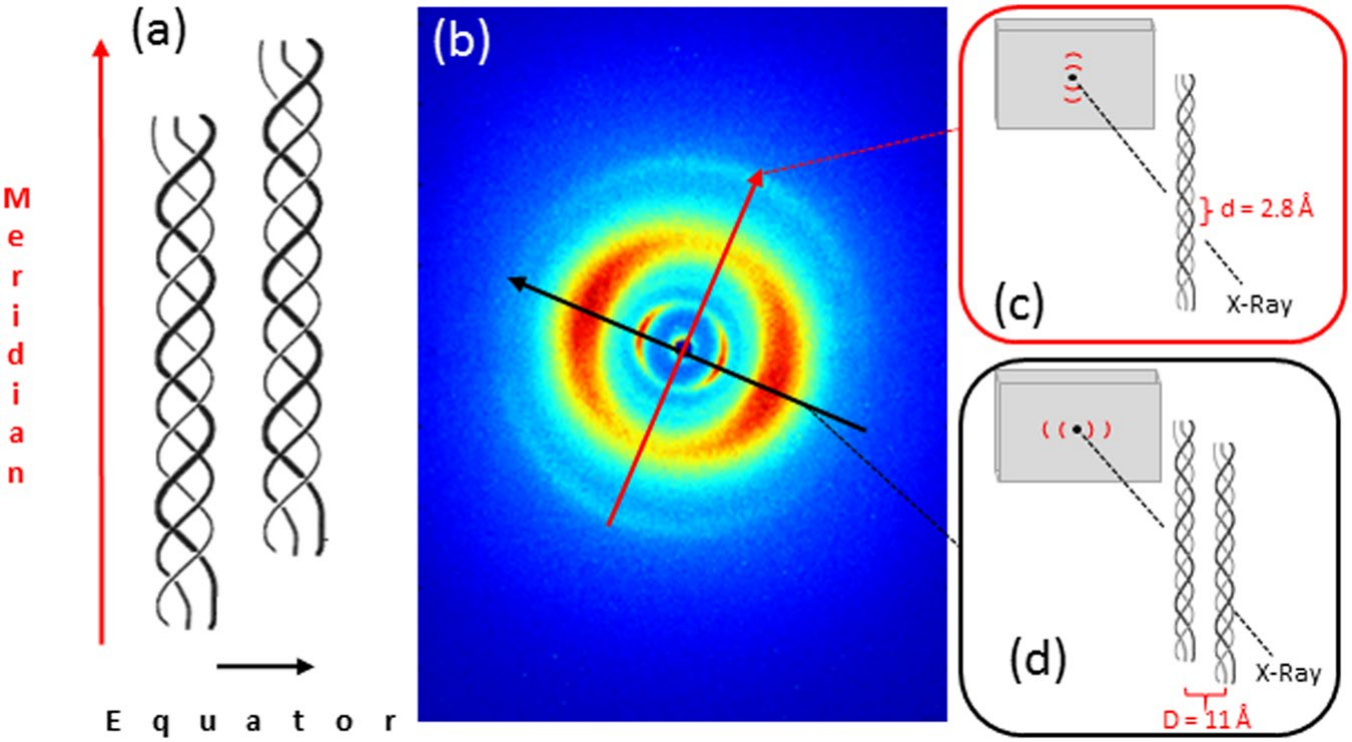
Diffracted intensities distribution of collagen triple helix (exemplificative WAXS data in b) obtained from equine tendon sample).

Wide angle X-ray reflections provide information about the collagen molecular structure along two principal directions: meridional and equatorial. The meridional reflections (red arrow in Fig. 1a,b) represent the electron density distribution along the central axis of helical structure, i.e. the distance between adjacent amino acid residues along triple helices (sketched in Fig. 1c), while the equatorial reflections (black arrow in Fig. 1a,b) represent the lateral packing of molecules inside the fibrillar structure (sketched in Fig. 1d).

As reported in the literature, there are two molecular models to describe the collagen triple helix: a triple stranded 10/3 helical model with a 10/1 helical symmetry of each peptide strand and a pitch length of 86 Å (Rich and Crick model), and a 7/2 helical conformation with a 7/1 helical strand symmetry and 60 Å pitch length.

The axial repeat of the collagen triple helices varies from 20.0 Å in 7/2 helix model (helical twist >51.4°) with 3.5 residues/turn, to 28.8 Å in 10/3 helix model with 3.33 residues/turn, depending on a high or low content of amino acids respectively^7,8^, despite a degree of variability in the helical twist along the length of the peptides can be observed.

These rod-like structures are assembled into fibrils (10–500 nm diameter), with intrafibrillar lateral packing that varies from 1.6 nm (wet bovine cornea samples) to 1.8 nm (dry rat tail tendon sample). Fibrils are further assembled into fibers with a typical packing distance ≥100 nm^9,10^.

Since it is well known that type I collagen is the primary determinant of mechanical properties of native tissues, several studies have focused on the relation between structural and mechanical features of collagen fibrils, in order to design functional artificial tissues^11^. However, little is known about the structural and mechanical correlation in collagen-based biomaterials or devices, typically having a collagen concentration lower than 40 mg/ml, which are widely used for cellular studies and/or *in vivo* applications. As a biomaterial, type I collagen is particularly advantageous as it directly stimulates cell adhesion and growth, by providing multiple cell attachment sites. The triple helical structure is selectively recognized by specific integrin receptors, such as α1β1, α2β1, α3β1, α10β1, and α11β1^12,13^, while additional binding sites, such as the RGD (Arg-Gly-Asp) ligand, may become available to cell attachment by triple helix unfolding. Moreover, collagen is non-immunogenic, has good hemostatic properties and regulates cell differentiation^14–16^. Collagen can also be processed in a large number of forms, such as films, gels, conduits or sponges, which make it highly versatile and suitable for the design of multiple biomedical devices, including tissue engineered medical products (TEMPs)^17^.

The device production usually starts with the dispersion of raw collagen flakes or fibers in aqueous solution, to obtain a collagen-based slurry suitable for further processing. It is worth stressing that all of the fabrication steps, going from slurry synthesis to shaping, crosslinking and sterilization of the intended device, may have a deep impact on the collagen structure and, as such, on the device performance. With specific focus on tissue engineering, where cell-material interactions are required to stimulate or enhance tissue regeneration, the structural changes induced by different processing conditions may also affect the way in which cells recognize and respond to functional collagen moieties. For example, collagen crosslinking, which is necessary to modulate the stiffness and the *in vivo* residence time of collagen-based scaffolds in order to match those required for tissue regeneration^18,19^, has been recently shown to impact also on cellular attachment^20,21^. In particular, Dehydrothermal (DHT) and Ethyl-3-(3-dimethylaminopropyl)carbodiimide)-based (EDC) zero-length crosslinking treatments, that are commonly adopted for collagen-based devices due to their high cytocompatibility, differently affect the availability of cell binding domains on the collagen molecules. If DHT may induce partial collagen denaturation^22^, thus contributing to the exposure of additional RGD ligands, EDC treatment may conversely impair cellular adhesion, by reducing the number of cell-adhesive sites on collagen triple helices.

While the effects of crosslinking on the properties of collagen-based scaffolds have been largely addressed, the potential influence of the collagen slurry production on the structural and biological properties of the final device is often neglected. Therefore, one of the goals of this work was to investigate whether and to what extent different protocols of slurry synthesis, for selected type I collagens available on the market, may change the protein structure at a molecular level and, as such, the final properties of the developed scaffolds. To this purpose, commercial type I collagens and collagen-based films, the latter prepared by air-drying of differently processed slurries and DHT/EDC crosslinking, were firstly characterized by Wide Angle X-ray Scattering (WAXS) technique, in order to inspect the characteristic structural features of both native materials and processed substrates, at the atomic scale. Further structural information at the molecular level was also acquired by Fourier Transform Infrared Spectroscopy (FT-IR). Then, tensile properties and proteolytic resistance of the films were investigated by means of uniaxial elongation tests and collagenase digestion assays, respectively, so as to correlate their mechanical performance and biodegradation rate to the structural changes induced by the processing.

Furthermore, believing that a deeper understanding of cell-collagen interactions is required for the optimization of collagen-based scaffolds and TEMPs, the immortalized RT4D6P2T rat schwannoma cell line was used as *in vitro* model to study the effects of the different slurry processing conditions on cell growth and differentiation. To the best of our knowledge, this is the first study that attempts to investigate the structural modifications of type I collagen resulting from different slurry processing conditions, and then to correlate them not only to the mechanical and degradation properties of collagen-based scaffolds, but also to the induced cellular response.

The choice of Schwann cells as preferred *in vitro* model was due to the fact that this work was originally directed to the optimization of type I collagen conduits and sponges for peripheral nerve regeneration^23,24^. As known in the literature, several collagen-based conduits, with variable porous structure and crosslinking, are currently available to reconnect the proximal and distal stumps of a transected peripheral nerve, in cases where direct suturing or nerve autograft are not feasible. Such conduits support the attachment and migration of myofibroblasts, thus limiting the formation of scar tissue at the site of injury, and also provide a protective chamber where Schwann cell migration and axon elongation can take place. However, the regenerative potential of the conduits dramatically drops as the gap length to be bridged increases. Practically, available devices can be used to bridge gaps up to a maximum of 3 cm^25^, while much larger defects are often encountered in the clinical practice. This clearly boosts the need for highly engineered, optimized conduits with enhanced regenerative capability.

Although inserted into the wide context of peripheral nerve regeneration, this work may be of great interest for the design and optimization of collagen-based scaffolds and TEMPs for various tissues and organs, due to the extensive and ubiquitous use of collagen in the tissue engineering and regenerative medicine fields, as well as for the production of efficient and customized substrates for cell differentiation.

## Results

### WAXS analysis of raw collagens

**Figure 2:**
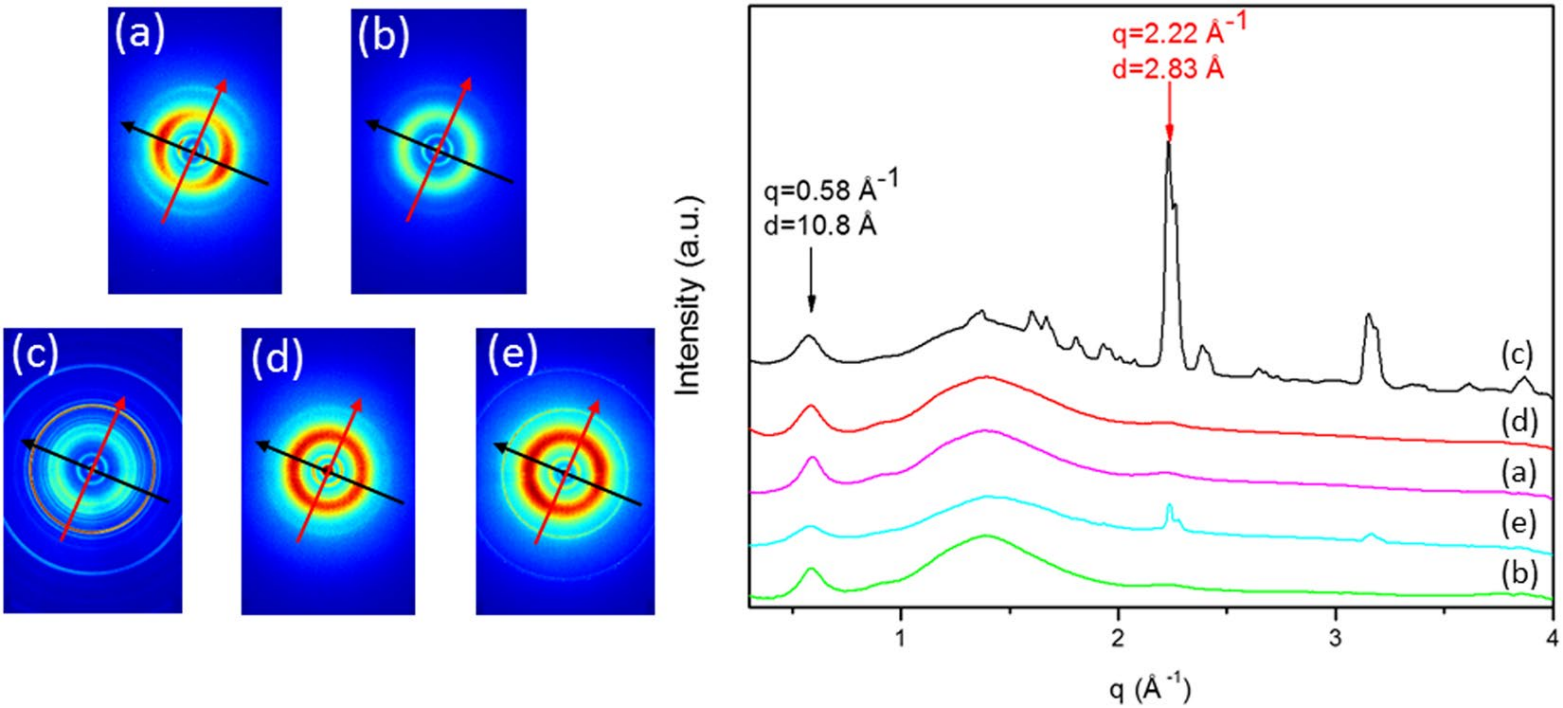
2D WAXS patterns, than folded into 1D WAXS profiles of raw type I collagens: Typ-ET-ch (**a**) and Typ-ET-en (**b**) derived from equine tendon, with the typical cross fiber diffraction pattern, and CS (**c**), SYM (**d**) and TYP-BH (**e**), derived from bovine dermis.

The analysis of the WAXS data and the plots of the 2D patterns were performed by the software SUNBIM^26^. 2D WAXS data collected on raw collagens (Fig. 2) show that collagen samples deriving from equine tendons (TYP-ET-ch and TYP-ET-en, Fig. 2a,b respectively) have the typical cross fiber diffraction pattern, clearly detectable in the chemically extracted sample (TYP-ET-ch, Fig. 2a). Both equatorial and meridional reflections are non-continuous rings, but arcs oriented along specific directions (preferred orientations marked by the two orthogonal arrows), with the maximum intensity along the black and red arrows, respectively.

On the contrary, 2D patterns of bovine dermis (Fig. 2c–e) show the presence of full rings without preferred orientation for both equatorial and meridional contributions. Moreover, additional diffraction rings are clearly visible in CS and TYP-BH patterns (Fig. 2c,e), due to salt contamination of the raw material (i.e. NaCl), related to the extraction processes. 2D patterns were then folded into 1D data profiles after the calibration procedure (Fig. 2).

From the analysis of the 1D profiles, two specific collagen reflections can be identified, as reported in the literature: the equatorial diffraction peak (q = 0.58 Å^−1^), that corresponds to an average apparent distance of 11 ± 2 Å between the triple helices in the quaternary structure (typical d-spacing of dry collagen molecules); and a weak and wide meridional diffraction peak (q = 2.22 Å^−1^), that corresponds to a distance of 2.8 ± 2.0 Å between adjacent amino acid residues along the central axis of helical structure. A broad band between 0.8 Å^−1^ and 2.1 Å^−1^ is also shown in Fig. 2, as the typical fingerprint of an amorphous-like contribution. Additionally, by comparing 1D data of raw collagens, salt contamination is more evident in the CS (black) than in the TYP-BH profile (cyan), since there are additional sharp peaks overlying the meridional one at around q = 2.22 Å^−1^.

Gaussian fitting of the equatorial peak, marker of intermolecular lateral packing, was then used to compute the full width at half maximum (FWHM), in order to evaluate the crystallinity degree of each sample (referring to the quaternary structure). Indeed, an increase in the structural disorder and consequent crystallinity loss are known to broaden the diffraction peaks^27^. As reported in Table 1, Type I collagen samples from bovine dermis (SYM, TYP-BH, CS) show wider peaks (amorphous-like profiles) compared to those collected from equine tendon (TYP-ET-ch, TYP-ET-en), thus indicating a lower degree of crystallinity of skin-derived collagen, compared to tendon one. Furthermore, FWHM exhibits a certain degree of variation among the different samples of bovine type I collagen. In particular, raw bovine collagen isoforms displayed decreasing levels of crystallinity according to the following order: SYM < CS < TYP-BH.

**Table 1:** WAXS crystallinity data (i.e. FWHM of equatorial diffraction peak) and evidence of preferred molecular orientation for raw collagen isoforms.

Raw collagen	FWHM (crystallinity)	PO (preferred orientation)
SYM	0.10 ± 0.003	NO
CS	0.11 ± 0.003	NO
TYP-BH	0.13 ± 0.003	NO
TYP-ET-ch	0.09 ± 0.003	YES
TYP-ET-en	0.09 ± 0.003	YES

Results are expressed as mean ± standard deviation.

### WAXS analysis of bovine collagen-based films

To evaluate the influence of various processing conditions on the collagen structure, bovine collagen isoforms (SYM, TYP-BH and CS) were differently dispersed in aqueous solution to obtain three different types of slurry. The corresponding films, obtained by air-drying and DHT/EDC crosslinking, were then characterized with Wide Angle X-ray Scattering technique. The combined zero-length crosslinking procedure (DHT/EDC) was adopted in view of cell culture studies, in order to confer proper enzymatic resistance to the collagen-based films without inducing cytotoxic effects. Although it is well known that DHT and EDC crosslinkings may induce some structural changes in collagen (e.g. concurrent partial denaturation for DHT crosslinking) all of the films were subjected to the same crosslinking treatment, so that any structural difference detected among them could be reasonably ascribed to the collagen source and/or the slurry processing.

**Figure 3:**
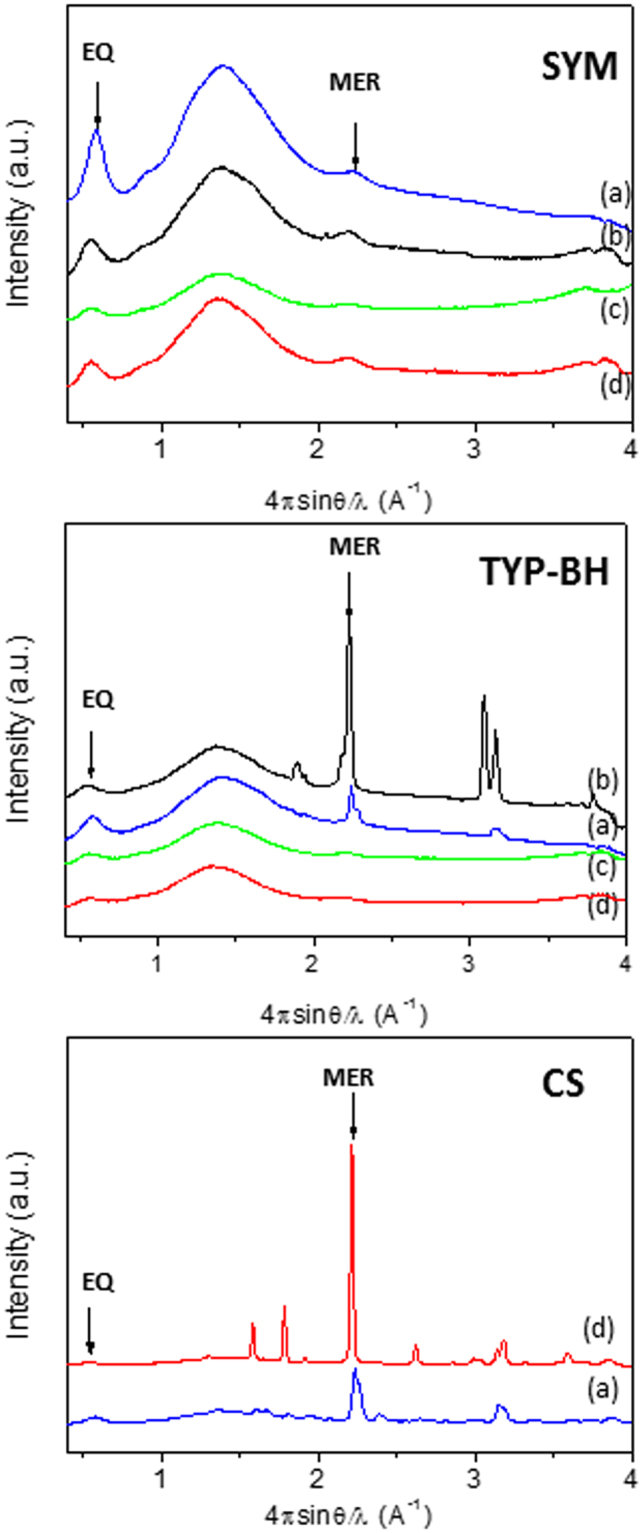
1D WAXS profiles of raw bovine collagen isoforms and processed collagen films: SYM, TYP-BH and CS. The raw material (**a**) is compared with the films obtained by different slurry processing treatments, i.e. HH (**b**), AA (**c**) and OMO (**d**) treatments. Equatorial and meridional peaks, signals of structural integrity, are marked by black arrows.

The 1D WAXS profiles of the collagen films are shown in Fig. 3 for SYM, TYP-BH and CS collagens, respectively. For each collagen isoform, the comparison of the raw collagen (a) flakes with the films made by HH (b), AA (c) and OMO treatments (d) is also reported. First of all, it is possible to notice that, for each collagen, the films display the same specific reflections found for the raw material, and a general decrease of salt contamination, as expected. However, the intensity and FWHM of the equatorial diffraction peak of the processed films (Fig. 3 and Table 2) change with respect to those recorded for the raw materials, thus highlighting that the processing conditions actually influence the order/disorder level in the lateral packing of the collagen molecules. In particular, a significant intensity decrease after acidic (AA) treatment could be noted for both SYM and TYP-BH collagens (green profiles in Fig. 3).

**Table 2:** Full width at half maximum (FWHM) measurements of the equatorial diffraction peak of raw bovine type I collagen isoforms and processed collagen films (mean ± standard deviation).

	PROCESSING	FWHM
SYM	Raw	0.10 ± 0.003
	HH	0.12 ± 0.003
	AA	0.13 ± 0.003
	OMO	0.12 ± 0.003
TYP-BH	Raw	0.13 ± 0.003
	HH	0.14 ± 0.003
	AA	0.13 ± 0.003
	OMO	0.12 ± 0.003
CS	Raw	0.11 ± 0.003
	OMO	0.10 ± 0.003

It is also worth noting that, for the SYM isoform (i.e. the one with the highest level of crystallinity in the raw state), the AA treatment concurs not only to the decrease of the equatorial peak intensity, but also to the increase of its FWHM (Table 2), with respect to the raw material and the other processing treatments (HH and OMO). If, on one side, this finding further confirms the higher quaternary structure disorder attained by the acidic treatment, it is very interesting to observe, on the other, the unexpected decrease of the FWHM value upon homogenization in acidic conditions (OMO vs. HH and AA treatments, in Table 2). Notably, such a decrease could also be detected for the TYP-BH isoform. In the specific case of TYP-BH collagen (i.e. the one with the lowest level of crystallinity in the raw state), the homogenized sample was found to display even a lower FWHM value (i.e. a higher level of structural order) than the raw material. With regard to CS collagen, which could be processed only by homogenization, the FWHM values of the equatorial diffraction peak of both raw material and processed films were quite comparable.

### FT-IR analysis

Collagen samples were also analyzed by means of FT-IR ATR spectroscopy, in order to obtain further information on the structural modifications of collagen related to different processing protocols, from a molecular point of view. FT-IR spectroscopy is indeed a powerful technique for the characterization of protein samples and is typically used for collagen-based materials^28–31^. Each protein is characterized by specific IR features which can be used to evaluate secondary structure organization, fibrillation, denaturation, and so on^32–34^. In particular, Amide I (ascribable to C=O stretching mode of the peptide bond) and Amide II (ascribable to N-H stretching mode of the peptide bond) are two typical IR signals of a protein that can be considered as marker bands and are located at about 1670–1620 cm^−1^ and 1570–1530 cm^−1^ respectively. Amide I was demonstrated to be related to the secondary structure of the protein and, in particular, of collagen^35–37^.

**Figure 4:**
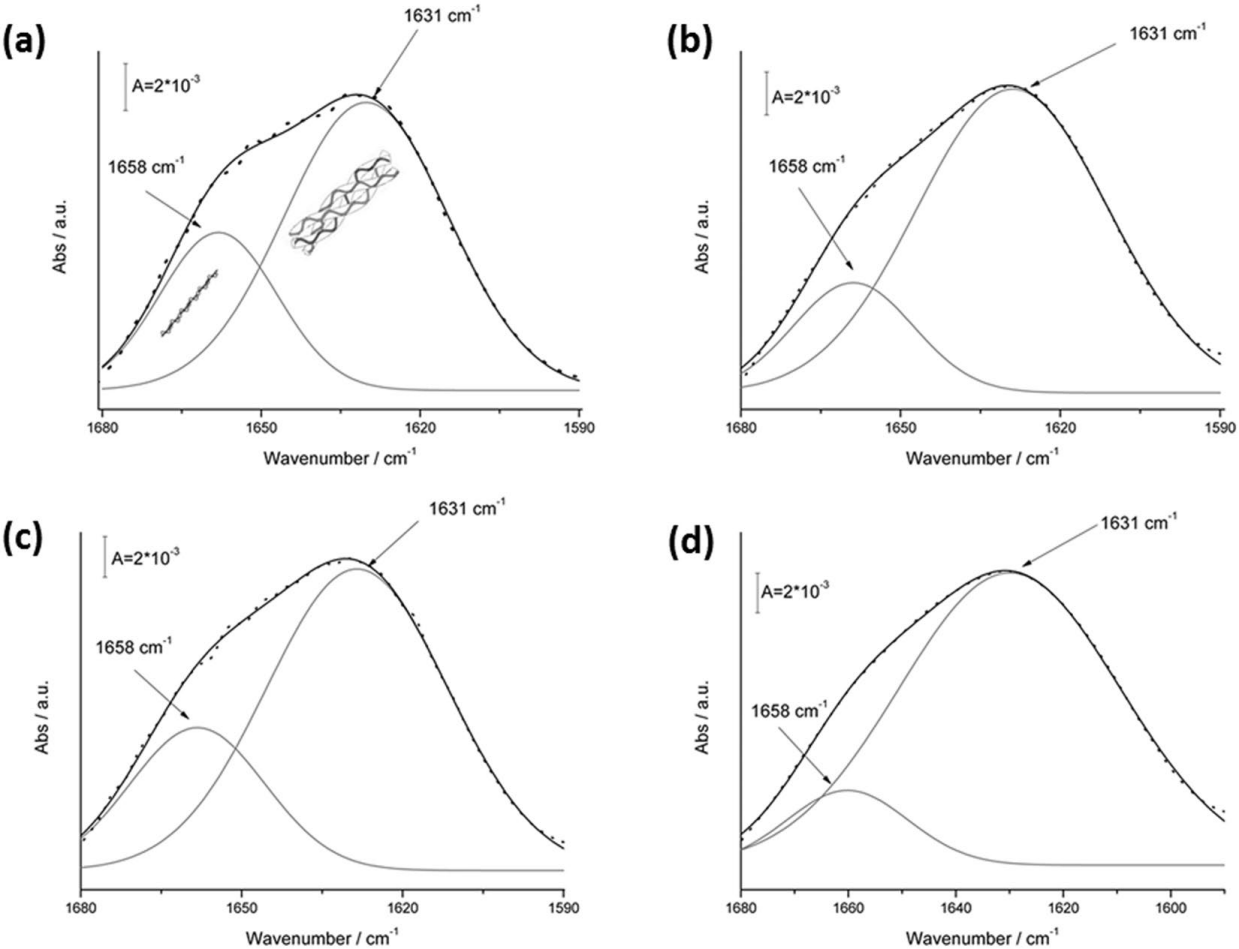
Amide I peak deconvolution (gray line) of α-helix and triple helix contributes (black line) elaborated by experimental curve (scatter plot) in TYP-BH collagen samples: raw material (**a**) and collagen-based films processed by HH (**b**), AA (**c**) and OMO (**d**) treatments.

Supplementary Figure S1 reports a typical FT-IR spectrum of collagen in the 3600–800 cm^−1^ frequency range. The spectrum is dominated by the amide I (at about 1631 cm^−1^), amide II (1550 cm^−1^) and amide III (1270 cm^−1^, ascribable to tertiary C-N stretching mode) bands. Collagen amide I is an asymmetrical band, due to the contributions of different secondary structure organizations. As shown in Fig. 4, deconvolution of the amide I band allows to identify two main peaks: a peak at 1631 cm^−1^, which can be ascribed to triple helix contribution, and a peak at 1658 cm^−1^, related to α-helical structure. In this study, deconvolution of the amide I band was thus exploited to evaluate the relative amount of triple helix depending on the different collagen isoforms and processing conditions under examination.

It was thus found that the processing procedure clearly affected the multiple structure of the collagen amide I band. Indeed, the 1631 cm^−1^/1658 cm^−1^ band ratio changed significantly, depending on the treatment. For example, in the case of TYP-BH collagen, the acidic treatment (Fig. 4c) induced a reduction of the triple helix organization, if compared with the other processing treatments. Conversely, the homogenization (Fig. 4d) seemed to be able to induce a strong triple helix organization.

The relative amount of triple helix (1631 cm^−1^) of both raw materials and processed films was calculated, for each collagen isoform and treatment (Table 3), as the percentage of the total amide I peak area ascribable to the triple helix peak, according to the following:

1 $$TH( \% )=100\ast \frac{{\int }_{1590\,c{m}^{-1}}^{1680\,c{m}^{-1}}(triple\,helix\,curve)d\bar{v}}{{\int }_{1590\,c{m}^{-1}}^{1680\,c{m}^{-1}}(amide\,I\,curve)d\bar{v}}$$

**Table 3:** The relative amount of triple helix (%) calculated by FT-IR spectroscopy for the investigated collagen-based samples.

COLLAGEN ISOFORM	PROCESSING	TH (%)
SYM	Raw	56.83
	HH	58.54
	AA	58.54
	OMO	61.29
TYP-BH	Raw	56.70
	HH	58.45
	AA	56.26
	OMO	60.98
CS	Raw	55.90
	OMO	55.90

The OMO treatment was found to show a strong amount of triple helix, for both TYP-BH and SYM samples. The triple helix percentage indeed increased in both cases to more than 60% of the total amide I contribution, compared to raw materials and other processing treatments.

It is also worth noting that for the TYP-BH isoform the AA treatment led to partial decrease of the triple helix amount, and an increasing the α-helical structure content (Fig. 4), while for SYM samples the AA treatment did not seem to affect the relative triple helix amount, compared to HH one (Table 3). In the case of CS collagen, which could be processed only by homogenization, the triple helix content remained unchanged compared to the raw sample.

### Mechanical tests

**Figure 5:**
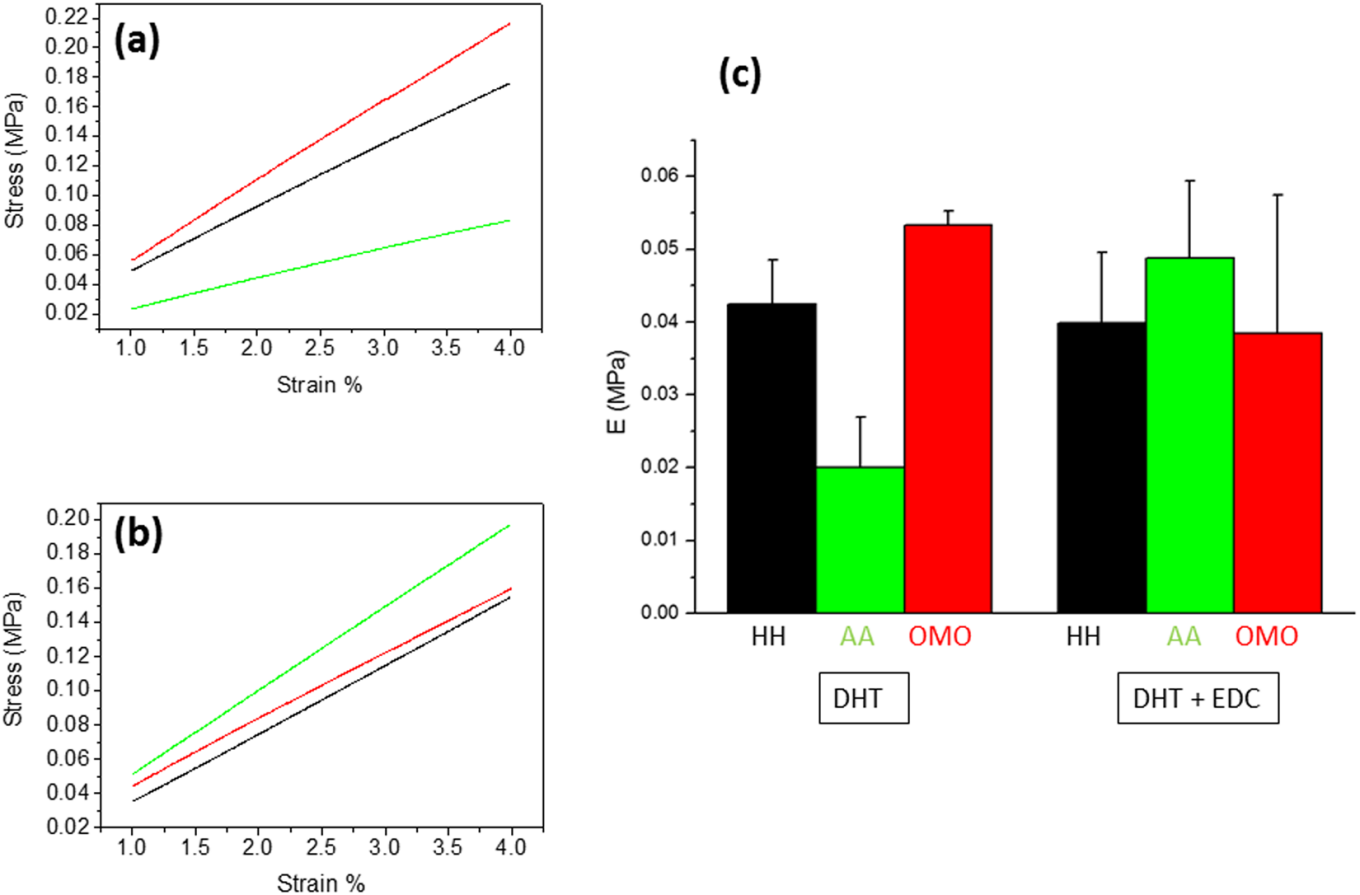
Evaluation of the elastic moduli of SYM films processed by HH (black), AA (green) and OMO (red) protocols, upon both DHT (**a**) and DHT/EDC (**b**) crosslinking. Histogram (**c**) directly compares the average moduli of the samples as a function of crosslinking treatment and processing. Error bars represent the standard deviation.

Mechanical characterization was done on the two collagen isoforms, CS and SYM, which had the minimum FWHM, i.e. the best crystalline degree, as reported in Table 2, in order to evaluate the influence of different collagen sources and processing conditions on the mechanical properties. In particular, since crosslinking is a key variable known to affect the stiffness of collagen-based devices, films crosslinked by combined DHT/EDC treatment were compared with those crosslinked by DHT alone, with the additional aim of verifying the efficacy of the double crosslinking method, in relation to the different collagens and processing conditions. As shown in Fig. 5 for SYM films, the Young’s modulus (E) was calculated from the stress-strain curves within the range 1–4% of material deformation, for both DHT and DHT/EDC films (Fig. 5a,b, respectively). Surprisingly, the results, summarized in Fig. 5c, showed that the combined DHT/EDC treatment significantly increased the stiffness of AA films (green bars), while HH and OMO samples (black and red bars, respectively) displayed similar E values upon both DHT and DHT/EDC treatments. The average reduction detected for the OMO samples upon combined DHT/EDC treatment was within the experimental error and was thus considered as not significant.

Moreover, it is interesting to observe that much higher E values were achieved by HH and OMO samples upon DHT crosslinking, compared to AA ones. Conversely, following DHT/EDC crosslinking, no significant differences could be detected among the differently treated samples.

With regard to CS films, that were processed only by homogenization (OMO), the elastic modulus was found to be 0.058 ± 0.010 MPa following DHT crosslinking and 0.192 ± 0.120 MPa following DHT/EDC one. For CS collagen films, the double crosslinking was thus found to lead to a significant increase of the stiffness, as expected.

### *In vitro* enzymatic degradation

The stability of collagen-based films was assessed *in vitro* by collagenase digestion tests that are widely used as an accelerated model of degradation. Collagenase recognizes the specific cleavage site -X-Gly- inside collagen peptide sequence –Pro-X-Gly-Pro-, where X is often a neutral amino acid. The BCA assay was used for the determination of collagen release during collagenase digestion.

While preliminary analysis on DHT-crosslinked films showed that all of the samples were rapidly and completely digested within 1 hour (data not shown), those crosslinked by double DHT/EDC treatment were found to be quite stable up to 2 hours of collagenase incubation, with a weight loss of approximately 20%. The films were then almost completely degraded after 4 hours. What is worth noting is that the degradation kinetics of all of the films tested appeared independent of the different collagen sources and processing conditions, as no significant differences could be detected among the samples.

### Cellular response

**Figure 6:**
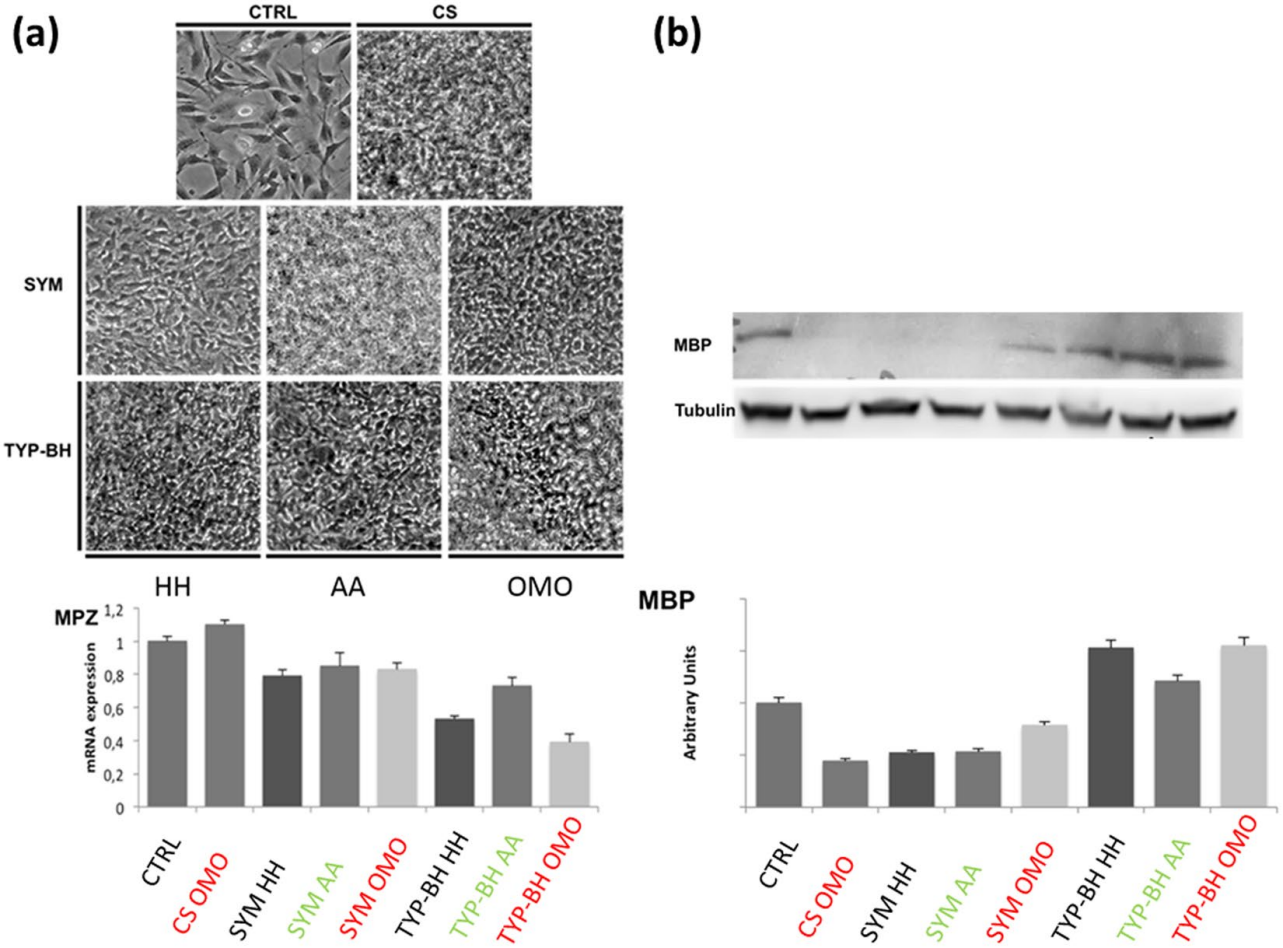
(**a**) Bright-field images of RT4D6P2T cells grown on different collagen-based films (upper panel) and mRNA expression levels of MPZ gene, as determined by relative real-time RT-PCR (lower panel); (**b**) protein expression levels of MBP as determined by Western blot.

Morphology of RT4D6P2T immortalized rat Schwann cells growing on the different collagen-based films produced in this study was observed by an inverse phase-contrast microscope (Fig. 6a), after 96 h of culture. Although in some samples (i.e.: CS, SYM AA and TYP-BH-OMO films) cells could not be efficiently focused and visualized due to the highly rough and irregular film surface in combination with high cell density, no significant differences were observed in cell morphology between cells cultured on the different collagen films and polystyrene plates (control). In all conditions, cells grew tightly on the different substrates keeping their regular star-like shape and contacting each other by projecting pseudopodia.

In order to investigate whether the different collagen films can affect the expression levels of Schwann cell specific markers, the MPZ mRNA levels and MBP protein levels were analyzed by Real Time RT-PCR and Western blot, respectively. The myelin associated protein MPZ is a major myelin protein and is one of the key markers for Schwann cell differentiation into myelinating cells. The different collagen films resulted to affect slightly MPZ mRNA expression (Fig. 6a, histogram). Nevertheless, the different isoforms of collagen seemed to influence the expression of MPZ, that was slightly down-regulated when cells were grown on SYM and TYP-BH films, with respect to the control.

On the other hand, the protein levels of the myelin-specific protein MBP were down-regulated in cells cultured on CS and SYM collagen films, while resulted up-regulated in Schwann cells grown on TYP-BH films. Interestingly, cell protein levels of MBP were higher when the films were treated by homogenization than in case of other treatments (Fig. 6b).

Overall, morphological and molecular investigation showed that both the specific type I collagen and the slurry processing method used to fabricate the collagen-based films can influence the behavior of RT4D6P2T cells, an *in vitro* model of Schwann cells.

## Discussion

WAXS data collected on raw materials showed the presence of collagen distinctive diffraction peaks, markers of helical structure and fibrillary packing preservation after extraction process, variable salt contamination and different triple helices orientation, depending on native tissues and extraction processes.

The presence of oriented signals along equatorial and meridional scattering directions in equine samples is the evidence of a preferential orientation of collagen molecules (Fig. 2a,b) inside the material, that is directly related to the spatial distribution inside native tissues; as it is well known, collagen fibers in tendons and ligaments are mainly oriented longitudinally, in order to transmit optimally forces of muscles and confer a great tensile strength^38^. Moreover, the FWHM evaluation of the equatorial peak (marker of intermolecular lateral packing) on raw materials demonstrated not only that equine type I collagen has a higher degree of crystallinity compared to bovine dermis samples, but also that there is a certain degree of variation among different samples of bovine type I collagen. This is the evidence of the influence of specific extraction process, in addition to the given tissue source, on the lateral interactions and packing of triple helices. In particular, raw bovine collagen isoforms displayed decreasing levels of crystallinity according to the following order: SYM < CS < TYP-BH.

It is worth noting that WAXS analysis performed on the corresponding films of bovine collagen, obtained by different slurry processing treatments (HH, AA, OMO) and zero-length crosslinking procedure, showed the influence of processing conditions on the intermolecular interaction of triple helices. In particular, after AA treatment not only did the intensity of equatorial diffraction peak decrease in both TYP-BH and SYM films, but also, and particularly in SYM, FWHM increased with respect to the raw material and the other processing treatments, suggesting that the protein quaternary structure was almost lost and the material had a lower order level of the collagen molecules after AA treatment.

On the contrary, in OMO conditions there was a decrease of FWHM value, thus suggesting that a higher crystalline domain of films, i.e. a higher degree of lateral organization of the triple helices, is generally attained upon mechanical homogenization of collagen suspensions, compared to magnetic stirring in distilled water or acidic solution. Interestingly, FT-IR data revealed that the OMO treatment also induced a strong increase of triple helix amount, for both TYP-BH and SYM films, to more than 60% of the total amide I contribution, compared to raw materials and other processing treatments. Notably, this increased amount of triple helix, together with the film’s increased crystalline domain or molecular packing shown by WAXS results (Table 2), indicates the formation of a highly ordered structure in homogenized samples. It is also worth highlighting that, while for the TYP-BH isoform the AA treatment led to partial disassembly of the triple helix, by increasing the α-helical structure content, for SYM samples the AA treatment did not seem to affect the relative triple helix amount, compared to HH one. These findings suggest that acidic processing conditions may differently influence the secondary protein structure, depending on the specific isoform tested.

With regard to CS collagen, which could be processed only by homogenization, WAXS data denoted a slight effect of the processing on the intermolecular interaction/order, in accordance with FT-IR findings of unchanged the triple helix content compared to the raw sample, suggesting no significant effect of the processing on the protein structure.

The mechanical tests performed on films showed that the combined DHT/EDC treatment significantly increased the stiffness of AA films, compared to HH and OMO samples, suggesting that the additional EDC crosslinking of the films, following the DHT treatment, was effective only when the raw SYM collagen was processed in acidic solution. This could be ascribed to the formation of a higher number of intermolecular, EDC-mediated zero-length crosslinks in AA samples, likely due to their increased structural disorder (i.e. lower crystallinity), as shown by WAXS analysis. On the contrary, the more ordered structure of HH and OMO samples (shown by both WAXS and FT-IR data) may have reduced the probability of additional zero-length bond formation (after DHT crosslinking) and/or facilitated the formation of intramolecular cross-links and loops, which typically do not contribute to elasticity^39^. In this regard, alternative, no zero-length crosslinking methods may be adopted to increase the elastically effective crosslink density of HH and OMO samples.

Moreover, it is interesting to observe that much higher E values were achieved by HH and OMO samples upon DHT crosslinking, compared to AA ones, suggesting that, as expected, the film stiffness was greatly affected by the structural packing of the collagen molecules, in addition to the crosslink density.

By comparing CS samples with similarly processed SYM ones (Fig. 5), it is evident that the collagen source deeply impacts the stiffness of the films, especially in case of double DHT/EDC crosslinking.

Unlike the mechanical stiffness, the specific mechanism of action of collagenase seemed insensitive to collagen source. The only variable acting on the degradation process seemed to be the crosslinking treatment, likely due to the slower diffusion of the enzyme in more crosslinked collagen networks.

On the contrary, different isoforms of collagen seemed to influence cellular behavior. In particular, the expression of MPZ (myelin associated protein) was slightly down-regulated when cells were grown on SYM and TYP-BH films with respect to the control.

Of particular note was that processing conditions also appeared to affect cell behavior. The protein expression of MBP was indeed up-regulated on samples processed by the OMO treatment, which was shown by WAXS and FTIR to induce the formation of ordered molecular structures.

## Conclusion

The aim of this work was the investigation of the structural features of different type I collagen isoforms at atomic and molecular scales, as well as the evaluation of the impact of different fabrication treatments on the structural, mechanical and biological properties of collagen-based films. WAXS data on raw materials demonstrated the preferential orientation of collagen molecules in equine tendon-derived collagens, either chemically or enzymatically extracted, while randomly oriented molecules were found in bovine skin collagens (SYM, TYP-BH and CS), together with a certain degree of salt contamination. The assessment of FWHM also allowed quantifying the crystallinity degree of the raw materials, related to the distance between adjacent molecules. WAXS and FT-IR analyses conducted on bovine collagen-based films, which were obtained through different slurry preparation protocols, interestingly showed that the OMO processing (i.e. mechanical homogenization of collagen slurry in acidic solution), expected to be the most aggressive processing condition, was actually the one ensuring a high content of super-organization of collagen into triple helices and a high crystalline domain into the material. Crosslinking of the films by combined DHT/EDC treatment was shown to affect their mechanical stiffness to a different extent, depending on the collagen source and the specific processing conditions. Conversely, collagenase-induced degradation kinetics appeared exclusively related to the crosslink treatment, without any detectable effect of the different collagens and fabrication methods tested. Moreover, with the ultimate aim of using the proposed collagens as substrates for peripheral nerve regeneration, *in vitro* tests on rat Schwannoma cells (RT4D6P2T) showed that Schwann cell differentiation into myelinating cells was dependent on the specific collagen film being used, and was found to be particularly stimulated in case of homogenization-treated samples. Although preliminary and specifically focused on nerve regeneration, these findings highlighted the importance of slurry processing conditions not only on the structural properties of collagen, but also on the cell response to the device being tested for tissue regeneration. This study may thus represent a useful basis for the future development or optimization of collagen-based scaffolds for multiple tissue engineering applications.

## Experimental section

### Materials

Several commercially available type I collagens were analyzed in this study, as specified in the following:

- Purified type I collagen from bovine hide, provided by Symatese Biomateriaux (Chaponost, France) and referred to as SYM.
- Fibrillar type I collagen from bovine hide, provided by Typeone Srl (Lecce, Italy) and referred to as TYP-BH.
- Fibrous type I collagen from bovine hide, provided by Collagen Solution Plc (Glasgow, UK) and referred to as CS.
- Type I collagen from equine tendon, provided by Typeone Srl (Lecce, Italy) and referred to as TYP-ET-ch.
- Purified type I collagen from equine tendon, provided by Typeone Srl (Lecce, Italy) and referred to as TYP-ET-en.

All other chemicals used in this work were purchased from Sigma-Aldrich, unless otherwise stated, and used as received.

### Methods

#### Collagen film preparation

Due to their versatile processing, bovine collagens (SYM, TYP-BH and CS) were selected for the preparation of collagen-based films, starting from aqueous collagen suspensions (also termed slurries). More in detail, with the aim of investigating the effect of different processing conditions on the structural properties of collagen, for each collagen isoform three protocols were adopted for the slurry/film synthesis. A first type of slurry (HH) was obtained by simply dispersing the selected amount of collagen fibers (2% w/v) in distilled water, by means of magnetic stirring for at least 4 hours (at 10 °C). A second slurry formulation (AA) was produced as described above, except that 0.5 M acetic acid was used as a solvent, instead of water. The third slurry type (OMO) was obtained by further homogenizing the AA suspension by means of an overhead blender (20 min, T = 10 °C, 7000 rpm). With regard to CS collagen, it was processed only by OMO treatment, since its fibers result insoluble in water or acidic solution. For the film preparation, all of the slurries were centrifuged at 7000 rpm for 10 min at 20 °C, to remove air bubbles, poured into Petri dishes and placed under a chemical hood to allow solvent evaporation.

The resulting films were finally peeled off and crosslinked by means of combined dehydrothermal (DHT) and carbodiimide (EDC) treatments, in order to stabilize the macromolecular network of collagen. DHT was carried out in a vacuum oven at 121 °C and 100 m Torr for 72 hours, as previously reported; additional EDC crosslinking was performed by immersing the DHT-treated samples in a crosslinking solution containing 6 m mol EDAC and 6 m mol NHS per g of collagen, according to a protocol described in the literature^40–42^. Films crosslinked only by DHT were also produced as a reference for mechanical and degradation tests.

#### X-Ray Scattering Measurements

Wide Angle X-ray Scattering (WAXS) experiments were performed at the X-ray Micro Imaging Laboratory (XMI-LAB)^43^ over raw collagens flakes and processed collagen-based films. The laboratory is equipped with a Fr-E + SuperBright rotating copper anode microsource (λ = 0.154 nm, 2475 W) coupled through a focusing multilayer optics Confocal Max-Flux (CMF 15–105) to a SAXS/WAXS (SWAXS) three pinhole camera equipped for X-ray scanning microscopy. An image plate (IP) detector (250 × 160 mm^2^, with 100 μm effective pixel size), with an off-line RAXIA reader, was employed to collect WAXS data. Raw collagen flakes and collagen-based films were directly mounted on the sample holder, and WAXS data were collected in three selected points per each sample. The spot size at the sample position was around 200 µm. The detector was placed at 10 cm distance from the sample, giving access to a range of scattering vector moduli (q = 4πsinϑ/λ) from 0.3 to around 3.5 Å^−1^, that correspond to 1.8–25 Å d-spacing range.

#### Infrared spectroscopy

Raw bovine collagens (SYM, TYP-BH and CS) and collagen based-films obtained by the different fabrication processes were analyzed by means of Fourier Transform Infrared (FT-IR) Spectroscopy in Attenuated Total Reflection (ATR) modality. In particular, spectra were obtained by using a PerkinElmer Spectrum One IR spectrometer in ATR mode with the resolution of 4 cm^−1^ and average scans of 64.

#### Mechanical characterization

Stress-strain tensile characterization was performed on collagen-based films, both DHT and DHT/EDC crosslinked, with the double aim of: (a) correlating the mechanical properties of the films to the different collagen sources and/or processing conditions being tested; and (b) verifying the expected stiffening effect of combined DHT/EDC crosslinking over DHT alone. Film samples (n = 5) of each type were cut in rectangular shape (1 cm × 3 cm) and soaked in PBS at room temperature overnight, before being subjected to uniaxial elongation by means of a Zwick-Roell mechanical tester. The hydrated sample thickness (about 0.15–0.20 mm) was measured by a Dino-Lite digital microscope. The films were then mounted between the clumps of the tester and immersed in a NaCl 0.9% (w/v) bath chamber. Each sample was initially pre-stretched with a load of 0.01 N. Thereafter, the movement of the crosshead was continued with a speed of 1 mm/min until rupture of the test sample^44^. Engineering stress and strain were calculated from the load-displacement data. Stress (σ) was calculated as

2 $$\sigma =\frac{F}{{A}_{0}}$$

where *F* is the force registered by the mechanical tester, while *A*_0_ is the initial cross-sectional area (width × thickness) of the gauge section of specimen. Strain (ε) was calculated as the ratio between the measured crosshead displacement (Δl) and the initial length (l_0_).

3 $$\varepsilon =\frac{\Delta l}{{l}_{0}}$$

The elastic or Young’s modulus (E), defined as the slope of the stress/strain curve at small strain, was elaborated within the range 1–4% of material deformation.

#### *In vitro* enzymatic degradation–BCA assay

The enzymatic stability of the collagen-based films was investigated in *vitro* by collagenase digestion tests.

Small pieces of each sample type (n = 3, 3 mg) were incubated at 37 °C in 3 ml of PBS Ca^2+^ Mg^2+^ (Buffer), containing 0.1 mg/ml of collagenase from *Clostridium Histolyticum*. 20 μl were taken from solutions at time points of 30 min, 1, 2 and 4 h, and stored at −20 °C. HH, AA and OMO films crosslinked only by DHT treatment were used as controls.

The bicinchoninic acid (BCA) assay was used for the determination of detectable levels of soluble collagen released during the collagenase digestion. BCA assay is based on the formation of a Cu^2+^ -protein complex (purple-blue), followed by its reduction and chelation in Cu^+^ ions. Cu^2+^ is reduced by specific amino acid residues (cysteine, tyrosine and tryptophan) and peptide bonds, therefore the reduction is proportional to the protein concentration. Calibration solutions were prepared using standard protein (BSA) in QuantiPro Working reagent solution (Reagent A: Reagent B, 25:1 and 1 part of Reagent C) at concentrations of 2.5, 5, 10, 15 μg/ml (QuantiPro BCA Kit Assay). The calibration standards were measured in duplicate and repeated for each analysis. Samples were placed into the wells of two 96-multiwell microplates. This was followed by the addition of 150 μl of QuantiPro Working reagent and then mixed. The plates were sealed and incubated at 37 °C for 2 h. The absorbance was then measured by a spectrophotometer at 562 nm.

#### Cell culture, real-time PCR and western blotting

To study the effect of different collagen sources and/or processing conditions on the cellular growth and differentiation the RT4D6P2T rat Schwannoma cell line was used as in vitro model. RT4D6P2T cells were grown in DMEM (Gibco) with 10% fetal bovine serum (FBS) (Sigma-Aldrich), 1% penicillin/streptomycin and 1% glutamine. Cells were seeded in 6-well culture plates at a density of 250, 000 cells/well directly on the different collagen-based films or on untreated polystyrene (control).

The mRNA and protein levels of the myelin specific genes Myelin Protein Zero (MPZ) and Myelin Basic Protein (MBP) were analyzed as markers of Schwann cell differentiation. For expression analysis of MPZ mRNA, total RNA was extracted using TRIzol® reagent (Life Technologies) and RNeasy plus mini kit (Qiagen), while RNA quantification was carried out with NanoDrop ND-1000 spectrophotometer. One microgram of RNA was retro-transcribed using iScript^TM^ select (Bio-Rad), and iQ^TM^ SYBR®green super mix (Bio-Rad) was used for Real-Time PCR. For analysis of protein levels of MBP, cultured cells were homogenized with a protein lysis buffer and protein quantification was performed using RC DC Assay kit (Bio-Rad). Protein lysates were separated by SDS-PAGE, transferred onto nitrocellulose membrane and incubated with primary antibody for MBP (Covance) and secondary antibody (Cell Signaling). Signal detection was performed using ECL plus reagent (Thermo Scientific), while image quantifications were carried out using ImageJ software (NIH).

## Electronic supplementary material

Supplementary Information
