# Supplementary material for: Effects of processing on structural, mechanical and biological properties of collagen-based substrates for regenerative medicine
Source: Sci Rep. 2018 Jan 23;8:1429. doi: 10.1038/s41598-018-19786-0 (PMC5780384; doi:10.1038/s41598-018-19786-0)
Supplement: Supplementary file 1 — Supplementary Information [file 41598_2018_19786_MOESM1_ESM.doc]

**SUPPLEMENTARY INFORMATION**

**Effects of processing on structural, mechanical and biological properties of collagen-based substrates for regenerative medicine**

A. Terzi^1^, E. Storelli^2,3^, S. Bettini^2^, T. Sibillano^1^, D. Altamura^1^, L. Salvatore^2^, M. Madaghiele^2^, A. Romano^3^, D. Siliqi^1^, M. Ladisa^1^, L. De Caro^1^, A. Quattrini^3^, L. Valli^4^, A. Sannino^2^ & C. Giannini^1^*

*^1^* Institute of Crystallography (IC), National Research Council, Bari, Italy

*^2^* Department of Engineering for Innovation, University of Salento, Lecce, Italy

^3^ Neuropathology Unit, Institute of Experimental Neurology and Division of Neuroscience, IRCCS San Raffaele Scientific Institute, Milan, Italy

^4^ Department of Biological and Environmental Sciences and Technologies, University of Salento, Lecce, Italy

*Correspondence and requests for materials should be addressed to [cinzia.giannini@ic.cnr.it](mailto:cinzia.giannini@ic.cnr.it)


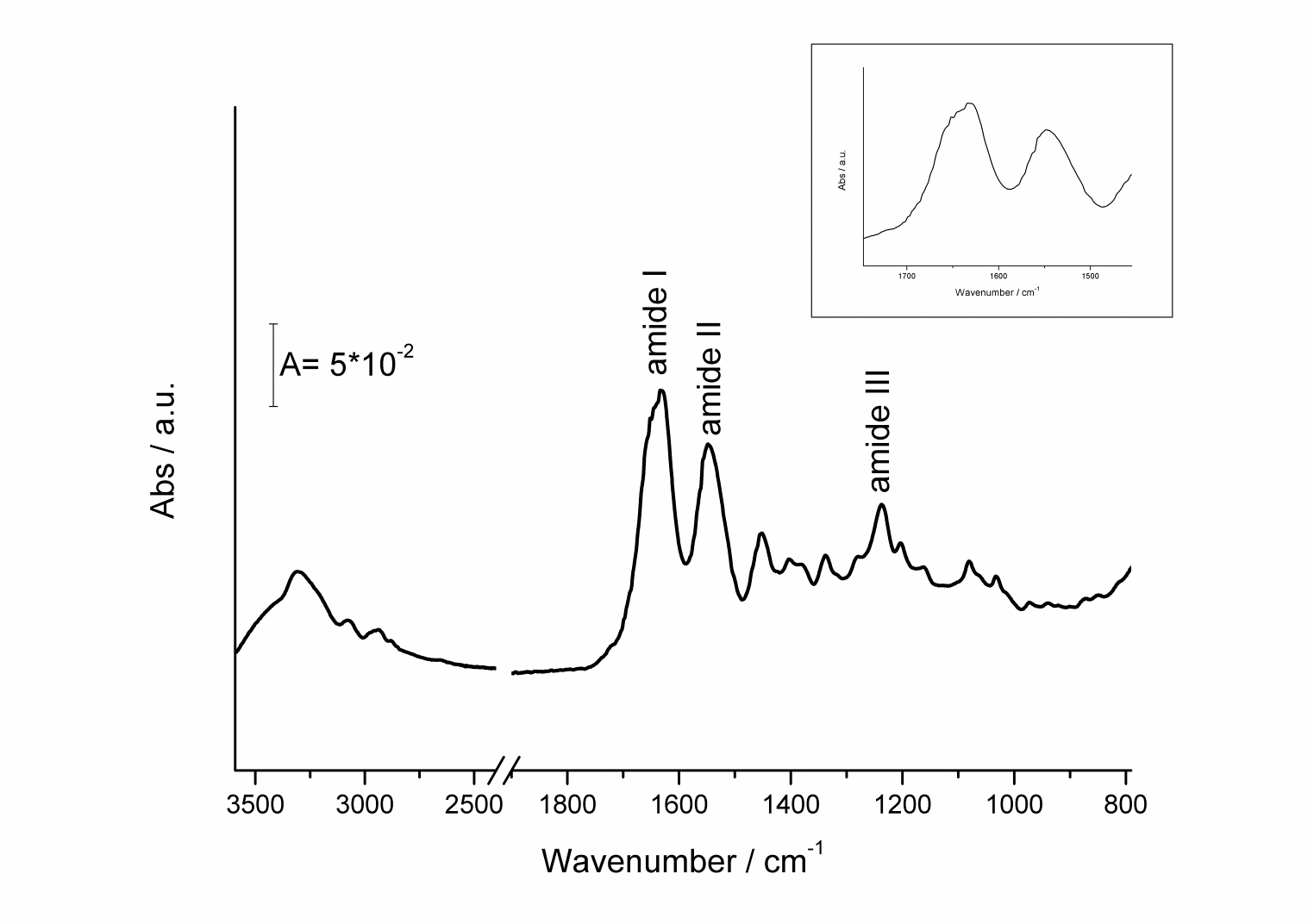


Supplementary Figure 1: FT-IR spectrum of Sym collagen raw samples in the 3600-800 cm-1 frequency range; in the inset, a magnification of the amide I and II region.


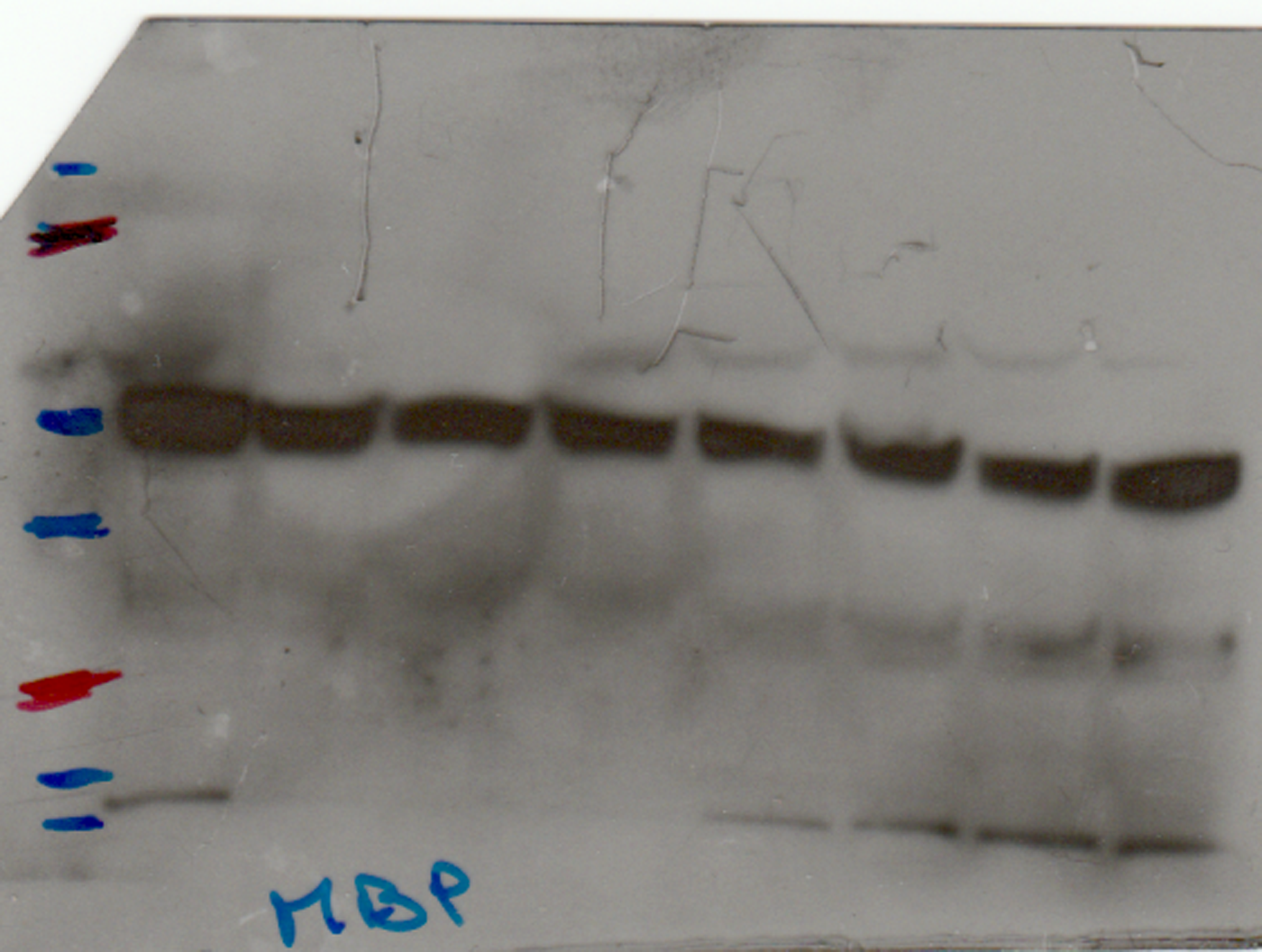


Supplementary Figure 2: Protein expression levels of MBP (20KD) in RT4D6P2T cells cultured on the different collagen-based films determined by Western blot. Marker highlight bands of 100, 75, 50, 37, 25, 20 and 15KD.
